# Occipital Nerve Stimulation for Refractory Occipital Neuralgia: A Multicenter, Randomized, Controlled Trial [StimO Study]

**DOI:** 10.3390/jcm15051922

**Published:** 2026-03-03

**Authors:** Stéphanie Ravaillault, Homaon Alipour, François Leger, Julien Labarre, Jean-Michel Nguyen, Yves Marie Pluchon, Thibault Riant, Évelyne Emery, Julien Nizard, Sylvie Raoul

**Affiliations:** 1 Department of Neurosurgery, UIC22, University Hospital, F-44000 Nantes, France; 2 University of Nantes, F-44000 Nantes, France; 3 Multidisciplinary Pain Center, Hôpital Yves Le Foll, F-22000 Saint Brieuc, France; 4 Multidisciplinary Pain Center, University Hospital, F-44000 Nantes, France; 5 Statistic Department CHU Nantes, F-44000 Nantes, France; 6 Multidisciplinary Pain Center, CHD La Roche sur Yon, F-44000 Nantes, France; 7 Multidisciplinary Pain Center, Hôpital Privé du Confluent Nantes, F-44000 Nantes, France; 8 Department of Neurosurgery, University Hospital, F-14000 Caen, France; 9 Nantes Université, Oniris, CHU Nantes, INSERM, Regenerative Medicine and Skeleton, RMeS, UMR 1229, F-44000 Nantes, France

**Keywords:** neuromodulation, occipital neuralgia, pain, peripheral nerve stimulation

## Abstract

**Introduction**: Occipital neuralgia [ON] is a primary headache disorder that contributes to a significant proportion of facial pain cases. Occipital neuralgia is an uncommon but disabling headache disorder with an estimated annual incidence of approximately 3.2 per 100,000 individuals. The pathophysiology of ON involves both sensitization of the greater occipital nerve and central mechanisms, including the sensitization of the trigeminocervical pain pathway. **Objective**: The aim of this study was to evaluate the effectiveness of occipital nerve stimulation (ONS) in patients with refractory occipital neuralgia after six months of treatment. **Materials and Methods**: StimO is a prospective, open-label, controlled, randomized, and parallel-group study comparing two groups: ONS (occipital nerve stimulation) and OMM (optimized medical management). **Results**: The percentage change in maximum pain between baseline (D0) and month 6 (M6) showed a significantly greater reduction (*p* = 0.04) in the ONS group compared to the OMM group. The EQ5D scores revealed that the ONS group had a better quality of life than the OMM group at month 1 (*p* = 0.01). Medication Quantification Scale (MQS) scores were significantly lower at M1, M3, and M6 [*p* = 0.03]. However, ONS did not significantly impact anxiety or depression, as assessed by the HAD scale [*p* > 0.05]. **Conclusions**: ONS appears to be safe and effective therapy, decreasing pain and medication use and improving quality of life at six months. **Trial Registration**: The study protocol is registered on ClinicalTrial.gov under the following registration number: NCT03475797

## 1. Introduction

### Background/Rationale

Occipital neuralgia (ON) is a primary headache disorder that accounts for 8.3% of facial pain cases [1]. Occipital neuralgia is an uncommon but disabling headache disorder, with an estimated annual incidence of approximately 3.2 per 100,000 individuals [2]. ON is the third most common headache syndrome, following migraine and tension-type headaches [3]. According to the International Headache Society (HIS), ON is defined as “Unilateral or bilateral paroxysmal, shooting, or stabbing pain in the posterior part of the scalp, in the distribution [s] of the greater, lesser, and/or third occipital nerves, sometimes accompanied by diminished sensation or dysesthesia in the affected area and commonly associated with tenderness over the involved nerve[s]” [4].

Management options include pharmacological therapy; local corticosteroid injections may alleviate pain but typically offer only temporary relief. If medical treatments fail, radiofrequency ablation of the greater occipital nerve can be considered, though there is a risk of pain recurrence over time. For cases where conventional methods are unsuccessful, more invasive techniques may be offered, such as C2 gangliotomy, C2 ganglionectomy, C2–C3 rhizotomy, C2–C3 root decompression, neurectomy, and neurolysis, with or without sectioning the inferior oblique muscle. These invasive procedures are becoming increasingly rare. Currently, there is growing interest in neuromodulation techniques, which are less invasive [5,6]. Occipital nerve stimulation (ONS) has been proposed as a treatment for various headache disorders, including migraine, cluster headache, other trigeminal autonomic cephalalgias, hemicrania continua, and ON [7].

The trigeminocervical pain pathway serves as the common pathophysiological basis for these headache disorders [8]. This pathway is involved in the sensory innervation and development of nociceptive pain in the head and neck [2]. The first-order neurons of various nociceptive signals [such as trigeminal neuralgia, migraines, and ON synapse with the trigeminocervical complex (TCC)]. The TCC includes the caudal part of the trigeminal nucleus [trigeminal nucleus caudalis at the brainstem level, as well as its extension into the upper cervical spinal cord (up to C2–C3)] [9]. The second-order neurons originating from the TCC project to the thalamus (specifically to its ventral posteromedial nucleus via the trigeminothalamic tract). Finally, the third neuron in the pain pathway is thalamocortical and projects to various regions of the brain [8]. Stimulation of the greater occipital nerve increases activity in the dorsal horn at C1 and C2, and subsequently in the trigeminal nucleus caudalis [10]. This is why occipital nerve stimulation (ONS) is a promising treatment for ON.

## 2. Materials and Methods

### 2.1. Objectives

The aim of this study was to assess the effectiveness and safety of ONS in patients with refractory ON after six months of treatment.

### 2.2. Study Design

The study lasted 54 months, with a 48-month recruitment period. Nine French centers participated in the study. The clinical trial sponsor was Nantes University Hospital, France. This was a prospective, open-label, controlled, randomized, and parallel-group study.

The primary outcome was to evaluate the efficacy of occipital nerve stimulation (ONS) in patients with refractory occipital neuralgia (ON) after six months of treatment. Success was defined as a reduction in pain of more than 50% after 6 months, compared with baseline, as assessed by a Visual Analog Scale (VAS).

Secondary outcomes included the following:

(a) Evaluation of the reduction in medical treatment after 6 months of treatment in the ONS group;
(b) Comparison between the two groups on several measures: maximum and average pain intensity over the previous 7 days, quality of life, patient’s overall impression of change, assessment after 6 months of treatment, and pain reduction at 3 and 6 months.

The following questionnaires were used: VAS MQS (Medical Quantification Scale), HAD (Hamilton Anxiety and Depression Scale), EQ-5D, and MIDAS.

### 2.3. Setting

Patients received treatment (ONS for occipital nerve stimulation or OMM for optimized medical treatment) for 6 months. In the OMM group, patients were followed for 6 months, while in the ONS group, patients were followed for 8 months (2 months between randomization and electrode implantation, followed by 6 months of follow-up). These two additional months were non-negotiable and allowed time to schedule the procedure in the operating room (including anesthesia consultation, equipment ordering, etc.).

**Figure 1:**
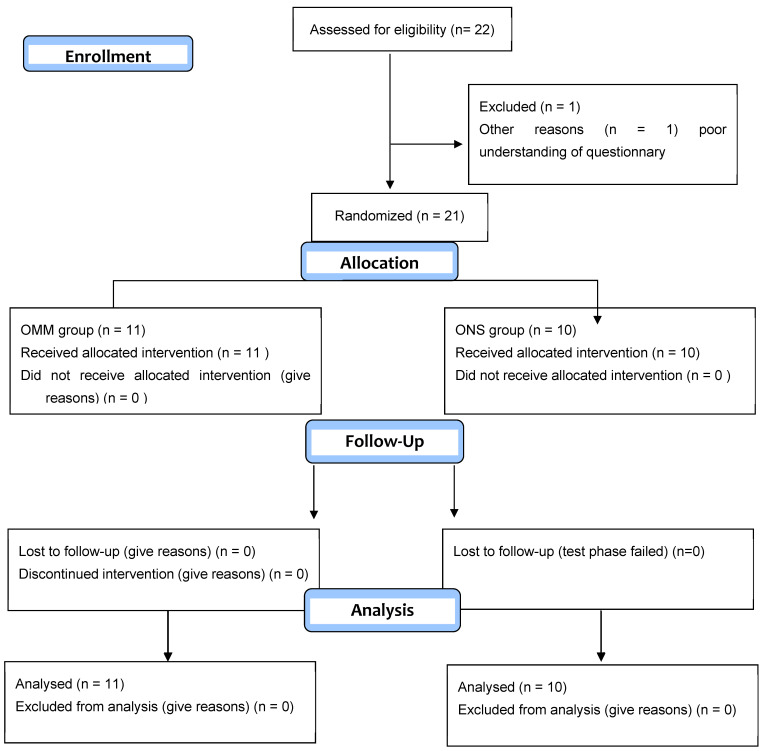
Flow diagram.

Four visits were planned (inclusion visit, and follow-up visits at 1, 3, and 6 months) (Figure 1). The inclusion visit included presenting the study and obtaining consent, verifying eligibility criteria, performing a pregnancy test [blood sample] for women of childbearing age, conducting clinical and neurological exams, a psychiatric evaluation [if not performed within the last 2 years], an interview with a psychologist, MRI of the skull and cervical spine [if not performed in the past 3 years], collection of current pain treatments, completion of pain and quality-of-life questionnaires, optimization of medical treatment by a pain physician, and randomization.

For patients randomized to the OMM group, the randomization day was Day 0. For patients randomized to the ONS group, consultations with an anesthesiologist and a pain physician were scheduled within the month of randomization. A first hospitalization (3–4 days) was planned for electrode implantation (Day 0). This hospitalization aimed to adjust stimulation parameters, train the patient to adjust stimulation, perform an X-ray to verify electrode positioning, and provide the patient with a logbook (to be completed over 7 days).

During the test phase, lasting at least 7 days [including a few days at home], pain was assessed and stimulation parameters were recorded. During the second hospitalization, the test phase was evaluated: if the test phase was positive, the equipment was permanently implanted, the patient was trained in using the remote control, and a logbook was provided [to be completed within 7 days prior to the next visit]. If the test phase was negative, the electrode was removed, a visit with a pain physician was scheduled, and the patient’s follow-up continued as part of the study. Treatment was adjusted in all cases.

At the 1-month, 3-month, and 6-month follow-up visits [after Day 0], the patient logbook [VAS, EQ-5D-5L, MIDAS, HAD, and list of medications] was collected, a new logbook with the same questionnaires was provided [except at the 6-month visit], adverse events were documented, pain treatments were adjusted, and stimulation parameters were adjusted [only for patients randomized to the ONS group].

### 2.4. Surgical Procedure

Depending on the practice of the surgeon, either a surgical lead (Lamitrode 4TM, St Jude Medical, Abbott 40/48 rue d’Arcueil Immeuble Miami 94593 Rungis Cedex, France) or a percutaneous lead (Quattrode 4^TM^, St Jude Medical) will be placed under local or general anesthesia in accordance to the practice of the center. Depending on whether the stimulation needs to be uni- or bi-lateral, single or dual extension will be used.

Depending on the intensity of the required stimulation, a pulse generator, either non rechargeable (Genesis) or rechargeable (Eon Mini), will be used. The decision to implant the subcutaneous generator (Eon Mini^TM^ or Genesis^TM^, St Jude Medical) is taken when the patient is considered, after a test phase (of at least 7 days), to respond to stimulation with 50% reduction of maximum pain and/or of average pain.

During the screening phase test, the stimulation is programmed by the physician, and then conducted at home with the possibility of another outpatient adjustment when considered necessary by the patient. The paresthesia must cover the whole painful territory, otherwise stimulation is ineffective. Pulse width was about 208 microseconds and 60 Hz, intensity was adjusted to be comfortable for the patient (range 1.2 mA to 3.8 mA, average 2.4 mA). Burst was not tested during the screening phase test.

The patient is readmitted to the hospital at the end of the one week test phase and an assessment is performed to evaluate the effect experienced:

- If the test is negative and/or if the patient refuses generator implantation, the electrode is removed.
- If the test is positive, the subcutaneous generator is implanted and the patient is instructed on the handling of the programmer.

All the surgeons performed occipital neuralgia stimulation for several years for cluster headache.

### 2.5. Participants

Inclusion criteria included:

- Patients aged between 18 and 85 years,
- Chronic ON lasting more than 6 months with persistent pain, with or without paroxysms [according to IHS criteria],
- Secondary ON [post-traumatic, post-surgical, due to major osteoarthritis, nerve compression or injury, etc.],
- Chronic neuropathic pain as per the Neuropathic Pain Diagnostic Questionnaire [DN4 ≥ 4],
- Maximum pain level [VAS] ≥ 50/100,
- Failure of medical treatments [combination of neuropathic pain medications such as antiepileptics and/or antidepressants and/or analgesics like paracetamol, tramadol, or morphine], and management in a Multidisciplinary Pain Center, including a multidisciplinary approach, physiotherapy, C1-C2 test block, radiofrequency rhizolysis and/or corticosteroid infiltration in C2, transcutaneous electrical neurostimulation [TENS] in the occipital region,
- Normal neurological examination, except in the occipital nerve territory,
- Negative pregnancy test for women of childbearing age, with effective contraception during the study,
- Patients capable of giving informed consent and having signed the informed consent form,
- Patients affiliated with a Social Security scheme.

Non-inclusion criteria included:

- Contraindication to the medical devices used, titanium allergies, contraindication to general anesthesia, complete anesthesia of the C2 territory [greater occipital nerve],
- Drug or alcohol dependence, psychiatric disorders [psychiatric evaluation required],
- Medical or psychological issues that could prevent the protocol from proceeding smoothly [e.g., cancer with limited life expectancy],
- Need for intensive nursing care, difficulty adhering to follow-up requirements,
- Pregnancy or breastfeeding, women refusing contraception, adults under legal guardianship, exclusion from other clinical trials, or participation in another clinical trial unless approved by the sponsor.

### 2.6. Study Size

The projected sample size was 70 patients, calculated with a 5% alpha risk and 80% power.

### 2.7. Statistical Methods

For the primary analysis, success was defined as a reduction of at least 50% in the Visual Analog Scale [VAS] score from baseline, assessed six months after randomization or at the last available assessment if the patient was lost to follow-up. The specific effect of each patient and the baseline pain level were accounted for by using relative change with paired measurements. A reduction of at least 50% from baseline (D0 to six months) was considered a success for the primary endpoint.

For patients who discontinued the study treatment for reasons other than death, every effort was made to continue VAS assessments. The last available VAS measurement was used for the primary efficacy analysis.

For the primary efficacy analysis, VAS scores were compared between groups A and B using Fisher’s exact test at a two-sided significance level of 5% in the modified Intention-To-Treat (ITTm) population. The ITTm population was defined as all randomized patients, excluding those who did not meet the eligibility criteria after randomization, those who did not receive the allocated treatment, and those for whom no clinical evaluations were available. If the null hypothesis was rejected, a 50% reduction in pain between D1 and M6 {[[D1 − M6]/D1] ≥ 50%} was compared between groups.

For secondary efficacy endpoints, assessment scales [VAS at 3 and 6 months, Medication Quantification Scale [MQS] at 3 and 6 months, Hospital Anxiety and Depression Scale [HAD] at 3 and 6 months, EQ-5D-5L at 3 and 6 months, and MIDAS at 3 and 6 months] were compared using the unpaired Wilcoxon test at a two-sided significance level of 5% in both the ITTm and Per-Protocol (PP) populations.

The placebo effect of the needle was also assessed. Pain was measured before needle implantation and again one day after implantation without any stimulation. The difference (D1 − D0) was compared between the randomized groups. No interim analysis was conducted.

All study activities were conducted under the approvals of CPP n° 17-NANT-01 (dated 19 March 2018) and ANSM (dated 26 December 2017) and with the full informed consent of all participants.

## 3. Results

The trial commenced on 20 April 2018 and included 22 participants, 21 of whom were randomized. Due to recruitment challenges during the COVID-19 period, the desired number of patients was not met. The original enrollment deadline of 20 April 2020 was extended to 20 April 2022.

By the reporting deadline, 21 patients had been randomized: 11 in the optimized medical management (OMM) group and 10 in the implantation and optimized treatment (ONS) group. In total, 11 patients from the OMM group and 9 patients from the ONS group completed the 6-month follow-up protocol [Figure 1].

### 3.1. Patient Characteristics

The patient characteristics are summarized in Table 1. The sex ratio was 7 male/15 female.

### 3.2. Efficacy

Regarding the primary endpoint (success at 6 months, defined as a reduction of at least 50% in pain between D0 and 6 months), no significant difference was found between the two groups. However, looking the percentage change between day 0 and 6 months, we observed a significantly greater decrease in average pain (*p* = 0.04) in the ONS group compared to the OMM group) but it is not 50%; it is about 44%. It is possible that with a few more patients the result could have been statistically significant. Pain as measured by average VAS was not significantly different between arms at Day 0, but it was significantly different between arms at 1 month and 3 and 6 months. Pain was significantly lower in the ONS group compared to the OMM group [Table 2].

However, a significantly greater reduction in maximum pain (*p* = 0.0441) was observed in the ONS group compared to the OMM group when measuring pain reduction between D0 and M6. If VAS at M6 was unavailable, the last available VAS measurement at M3 or M1 was used. Additionally, patients in the OMM group who were unsatisfied with the treatment after 6 months were switched to the ONS group. These patients showed a reduction in ON pain after transitioning to ONS. All patient except one (*n* = 10) were implanted. Data were collected with the same procedure (VAS, EQ5D Midas, and HAD). The results will be reported soon with a follow up of for years. None of the patients have asked to remove the stimulation.

**Figure 2:**
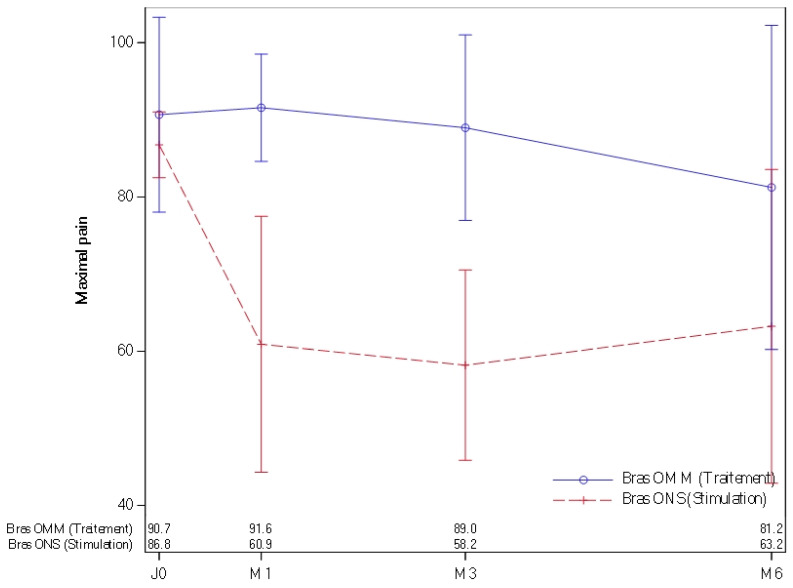
Maximal pain at 0, 1 month, 3 months, and 6 months in OMM patients [blue] and ONS patients (red).

Regarding secondary outcomes, maximum pain, measured by VAS, showed a significant difference between the groups at D0, M1, M3, and M6, with the ONS group reporting significantly lower pain levels compared to the OMM group [Figure 2]. Average pain levels were not significantly different between groups at D0, but they were significantly different at M1, M3, and M6 [Figure 2].

The EQ-5D score was significantly different between groups at M1, with the ONS group showing a better quality of life compared to the OMM group. However, no significant differences were found at D0, M3, and M6.

**Figure 3:**
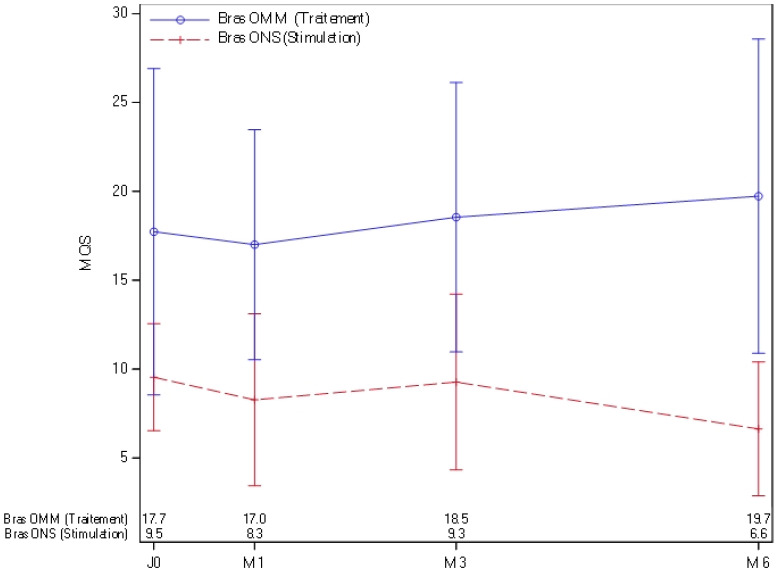
MQS at 0, 1 month, 3 months, and 6 months in OMM patients [blue] and ONS patients [red].

The total MQS score was significantly different between groups at M1, M3, and M6 (*p* < 0.05), with the ONS group showing lower scores than the OMM group [Figure 3].

There were no significant differences in HAD anxiety or depression scores between the groups at any follow-up time point.

**Figure 4:**
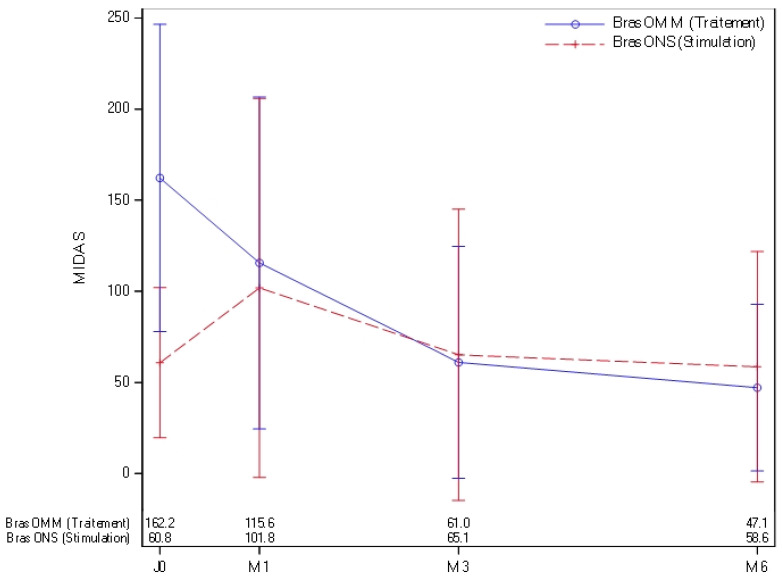
Midas score at 0, 1 month, 3 months, and 6 months in OMM patients [blue] and ONS patients [red].

The MIDAS score was significantly lower in the ONS group compared to the OMM group at D0 (median: 44.5 vs. 125.0), but no significant differences were observed at M1, M3, and M6 [Figure 4].

Two serious adverse events were reported. One patient in the ONS group developed a device-related site infection, requiring the removal of the implant, but the device was successfully reimplanted five months later. The second SAE involved a patient in the OMM group who was prescribed amitriptyline, topiramate, tramadol, and sumatriptan for occipital neuralgia and cluster headaches. After a sumatriptan injection, the patient experienced chest pain, elevated troponin levels, and ST elevation on ECG, though their condition improved. Non-serious adverse events were also reported.

## 4. Discussion

Although continuous measures of pain reduction favored ONS, the predefined primary endpoint was not met. Therefore, the present study does not strongly demonstrate superiority of ONS over optimized medical management. Nevertheless, there seems to be a superiority of the ONS treatment over medical treatment in intensity of pain, especially in reducing drug treatment and quality of life. To our knowledge, this is the first prospective study described in the literature. Pain as measured by average VAS was not significantly different between arms at Day 0; it was significantly different between arm at 1 month and 3 and 6 months. Pain was significantly lower in the ONS group compared to the OMM group. Indeed, one of the biases in this study regarding the failure to achieve the primary objective lies in the fact that pain increases at 6 months, either due to tolerance phenomena or because the drug treatment is reduced

Our findings align with previously published results. Weiner was among the first to report the efficacy of ONS in ON, showing that 12 out of 13 patients experienced significant pain relief [≥50% reduction in numeric rating scale [NRS] scores following ONS implantation] [2,11]. Similarly, Kapural observed excellent outcomes in a cohort of six patients, with 100% achieving at least a 50% reduction in pain at three months post-implantation. Additionally, the Pain Disability Index [PDI] showed significant improvement three months after the procedure [2,12]. In 2005, Nguyen et al. reported a reduction in the frequency of painful attacks by more than 50% in 91% (20 out of 22) of patients implanted with ONS. Furthermore, a decrease of at least three points in VAS scores was observed in 15 patients (70%). However, the cohort was heterogeneous, including 19 patients with cervicogenic headache or ON, one patient with typical cluster headache, and one patient with atypical cluster headache [13]. Slavin et al. described continued pain improvement in 7 out of 10 patients at an average follow-up of 22 months [14].

In the preliminary study that established the required sample size for our trial, ONS appeared to have a greater effect compared to OMM than what was ultimately observed in the randomized controlled trial. It is possible that a larger sample size would have yielded statistically stronger results. Nevertheless, despite recruitment challenges due to the COVID-19 pandemic, our findings still indicate a trend toward superior pain relief with ONS compared to OMM.

Regarding complications, Schwedt et al. (2007) reported a high rate of lead migration, affecting 60% of patients at two years post-implantation and 100% at three years [15,16]. Lead migration was also the most frequently reported adverse event in the ONSTIM feasibility study, occurring in 24% of cases [16,17]. In contrast, our study did not observe any cases of lead migration. This may be attributed to the shorter follow-up period [six months] and advancements in electrode design since 2007, allowing for better anchoring, as well as improvements in surgical techniques, such as creating loops to reduce excessive traction. Other potential complications include device malfunction, inappropriate or excessive stimulation, device shock, lead erosion, infections, lead fractures, and lead disconnections. According to the MAUDE database, infections account for 155 of the 1233 reported adverse events related to ONS [18], and infections may affect up to 30% of implanted patients [16]. In our study, only one patient experienced a device infection despite systematic antibiotic prophylaxis.

Regarding alternative treatments for ON, Kapoor et al. [19] demonstrated the efficacy of CT fluoroscopy-guided percutaneous C2-C3 nerve blocks, providing complete pain relief in all patients for at least one to four hours after bupivacaine infiltration. However, these results highlight the transient nature of this technique. In the same study, patients with a positive response to nerve blocks underwent dorsal rhizotomy, involving sectioning of the C1, C2, C3, and upper C4 dorsal nerve rootlets on the affected side. After an average follow-up of 20 months, 64.7% of the 17 patients reported complete symptom relief, 11.8% had partial relief, and 23.5% experienced no improvement. No serious adverse effects were reported [19]. Dubuisson et al. also reported excellent outcomes with microsurgical dorsal root entry zone [DREZ] lesions on C1, C2, and upper C3 dorsal rootlets. Seven out of 14 patients were pain-free and medication-free, while three achieved more than 50% pain relief. The remaining four patients experienced over 50% relief from occipital pain, though temporal and periorbital pain persisted. The average follow-up was 33 months [range: 3–66 months] [20]. Like ONS, rhizotomy appears to provide long-term efficacy in most patients, but these techniques are more invasive and associated with higher risks of postoperative complications.

Despite being a controlled and randomized study, our trial has certain limitations. First, since ONS is already an established treatment, we could not compare it to a placebo, which would have allowed for a more precise assessment of its true efficacy. Second, there is an evaluation bias, as the assessment of clinical outcomes could not be blinded due to the surgical procedure. Finally, our sample size was limited to 21 patients instead of the planned 70, which of course reduces statistical power. A larger randomized study is necessary to confirm and further explore the impact of ONS.

Lastly, we question the relevance of the VAS scale for assessing chronic pain. While it is widely used in clinical practice and demonstrates excellent intra-rater reliability [21], its ability to comprehensively evaluate chronic pain has been challenged. VAS scores primarily capture pain intensity, but chronic pain is multifaceted and extends beyond intensity alone. This limitation has led to the development of alternative tools that assess broader aspects of pain, such as its impact on daily activities and quality of life [EQ-5D-5L], mental health [Hospital Anxiety and Depression Scale [HAD]], and medication consumption [Medication Quantification Scale [MQS]] [22].

## 5. Conclusions

ONS is a safe and effective therapy that appears to reduce the percentage change in maximal pain between D0 and M6. Regarding secondary endpoints, the EQ-5D results indicate that the ONS group had a better quality of life than the OMM group at M1. Additionally, ONS seems to reduce drug consumption at M1, M3, and M6. However, it does not appear to have a significant effect on anxiety and depression scores as measured by the HAD scale. Similarly, no changes were observed in the MIDAS score at M1, M3, or M6.

Given these findings, ONS could appears to be an effective therapeutic option for patients suffering from occipital neuralgia who are refractory to conventional treatments. The trial should be regarded as exploratory due to recruitment limitations. However, further studies with a larger sample size are needed to confirm these results and strengthen the evidence supporting its efficacy.

## Figures and Tables

**Table 1:** Patients’ characteristics.

		Bras OMM *n* = 11	Bras ONS *n* = 11	Total *n* = 22
Age	*n*	11	11	22
	Min-Max	[26.00; 66.00]	[29.00; 69.00]	[26.00; 69.00]
	Moyenne+/−Ecart-type	49.09+/−13.02	45.55+/−12.35	47.32+/−12.51
	Médiane [Q1;Q3]	52.00 [37.00; 59.00]	45.00 [34.00; 50.00]	49.00 [37.00; 58.00]
*Age du patient*				
Sexe	*n*	11	11	22
	Masculin	5 [45.45%]	2 [18.18%]	7 [31.82%]
	Féminin	6 [54.55%]	9 [81.82%]	15 [68.18%]
*Sexe du patient*				
Neurological exam	*n*	11	11	22
	Normal	11 [100.00%]	11 [100.00%]	22 [100.00%]
	Abnormal	0 [0.00%]	0 [0.00%]	0 [0.00%]
*Examen neurologique*				
Clinical score: Score DN4	*n*	11	11	22
	Min-Max	[5.00;10.00]	[4.00;8.00]	[4.00;10.00]
	Moyenne+/−Ecart-type	6.73+/−1.68	6.00+/−1.48	6.36+/−1.59
	Médiane [Q1;Q3]	6.00 [5.00; 8.00]	6.00 [4.00; 7.00]	6.00 [5.00; 7.00]
*Examen clinique: Score DN4*				
Psychologist evaluation	*n*	11	11	22
	Non fait	0 [0.00%]	0 [0.00%]	0 [0.00%]
	Fait	11 [100.00%]	11 [100.00%]	22 [100.00%]
*Entretien avec un psychologue*				
*Raison de la non-réalisation de l’entretien*				
Localisation of occipital pain	*n*	11	11	22
	Strictly unilatéral	5 [45.45%]	8 [72.73%]	13 [59.09%]
	Preferentially unilatéral	2 [18.18%]	0 [0.00%]	2 [9.09%]
	Bilatéral	4 [36.36%]	3 [27.27%]	7 [31.82%]
*Localisation des douleurs occipitales*				
Pain side	*n* missed	4	3	7
	*n*	7	8	15
	right	3 [42.86%]	2 [25.00%]	5 [33.33%]
	left	4 [57.14%]	6 [75.00%]	10 [66.67%]
*Douleur unilatérale: Côté*				
Acute pain	*n* missed	1	0	1
	*n*	10	11	21
	NON	2 [20.00%]	0 [0.00%]	2 [9.52%]
	OUI	8 [80.00%]	11 [100.00%]	19 [90.48%]
*Crise aigue*				
Number of crisis for 1 month	*n* missed	3	0	3
	*n*	8	11	19
	Min-Max	[6.00; 60.00]	[1.00; 30.00]	[1.00; 60.00]
	Moyenne+/−Ecart-type	28.50+/−17.88	14.00+/−13.22	20.11+/−16.60
	Médiane [Q1;Q3]	30.00 [14.00; 37.00]	6.00 [2.00; 30.00]	20.00 [5.00; 30.00]
*Nombre de crise sur 1 mois*				
Professionnal activity	*n*	11	11	22
	NO	6 [54.55%]	5 [45.45%]	11 [50.00%]
	YES	5 [45.45%]	6 [54.55%]	11 [50.00%]
*Activité professionnelle*				
Profession	*n* missing	6	5	11
	*n*	5	6	11
	EDF agent	1 [20.00%]	0 [0.00%]	1 [9.09%]
	Maintenance worker	1 [20.00%]	0 [0.00%]	1 [9.09%]
	Farmer	1 [20.00%]	0 [0.00%]	1 [9.09%]
	Technical sales assistant	0 [0.00%]	1 [16.67%]	1 [9.09%]
	Driver	0 [0.00%]	1 [16.67%]	1 [9.09%]
	Multi-tasking sale manager	1 [20.00%]	0 [0.00%]	1 [9.09%]
	Customer advisor in a bank	1 [20.00%]	0 [0.00%]	1 [9.09%]
	Insurance claim advisor	0 [0.00%]	1 [16.67%]	1 [9.09%]
	Building painter	0 [0.00%]	1 [16.67%]	1 [9.09%]
	Medical secretary	0 [0.00%]	1 [16.67%]	1 [9.09%]
	Security guard	0 [0.00%]	1 [16.67%]	1 [9.09%]
*Profession*				
Current professional activity	*n* missing	6	4	10
	*n*	5	7	12
	NO	3 [60.00%]	2 [28.57%]	5 [41.67%]
	YES	2 [40.00%]	5 [71.43%]	7 [58.33%]
*Activité professionnelle en cours*				
Work stoppage	*n* missing	8	9	17
	*n*	3	2	5
	NO	1 [33.33%]	1 [50.00%]	2 [40.00%]
	YES	2 [66.67%]	1 [50.00%]	3 [60.00%]
*Arrêt de travail*				
Work accident	*n* missing	8	9	17
	*n*	3	2	5
	NO	2 [66.67%]	2 [100.00%]	4 [80.00%]
	YES	1 [33.33%]	0 [0.00%]	1 [20.00%]
*Accident du travail*				
Disability due to pain	*n* missing	6	4	10
	*n*	5	7	12
	NO	5 [100.00%]	7 [100.00%]	12 [100.00%]
	YES	0 [0.00%]	0 [0.00%]	0 [0.00%]
*Invalidité du fait de la douleur*				
Type of disability	*n* Missing	11	11	22
	Total disability	0 [0.00%]	0 [0.00%]	0 [0.00%]
	Partial disability	0 [0.00%]	0 [0.00%]	0 [0.00%]
*Type d’invalidité*				
*Catégorie d’invalidité*				
Medical and surgical antecedent	*n*	11	11	22
	NO	0 [0.00%]	0 [0.00%]	0 [0.00%]
	YES	11 [100.00%]	11 [100.00%]	22 [100.00%]
*Présence d’antécédents*				

**Table 2:** Average VAS in ONS and OMM group.

		Bras OMM *n* = 11	Bras ONS *n* = 9	Total *n* = 21	*p*-Value
Average VAS—J0	*n* missing	1	0	1	
	Médiane [Q1;Q3]	74.00 [53.00; 90.00]	65.00 [53.00; 68.00]	65.00 [53.00; 74.00]	0.2448
EVA moyenne—J0					0.2448
Average VAS—M1	Médiane [Q1;Q3]	80.00 [58.00; 87.00]	29.00 [17.00; 48.00]	50.50 [29.00; 80.00]	0.0002
EVA moyenn —M1					0.0002
Average VAS—M3	*n* manquant	1	1	2	
	Médiane [Q1;Q3]	79.50 [57.00; 89.00]	29.50 [19.00; 49.00]	53.00 [29.50; 79.50]	0.0007
EVA moyenn —M3					0.0007
Average VAS—M6	*n* manquant	0	2	2	
	Médiane [Q1;Q3]	72.00 [45.00; 85.00]	40.00 [24.00; 50.00]	56.00 [32.00; 73.50]	0.0225
EVA moyenne—M6					

## Data Availability

The original contributions presented in this study are included in the article. Further inquiries can be directed to the corresponding author.

## Abbreviations

The following abbreviations are used in this manuscript:

ON	occipital neuralgia
ONS	occipital nerve stimulation
OMM	optimized medical management
NRS	numeric rating scale
MQS	Medical Quantification Scale
HAD	Hamilton Anxiety and Depression Scale
